# Nanoscale Insights into the Dynamics of Conductive Filament Growth/Dissolution in 2D Material‐Based Memristors

**DOI:** 10.1002/advs.202509791

**Published:** 2025-10-14

**Authors:** Chen Li, Rui Pan, Tao Xu, Jiaxin Shen, Yatong Zhu, Mingrui Zhou, Xiaohui Hu, Kuibo Yin, Litao Sun

**Affiliations:** ^1^ SEU‐FEI Nano‐Pico Center Key Laboratory of MEMS of Ministry of Education Southeast University Nanjing 210096 China; ^2^ College of Materials Science and Engineering Nanjing Tech University Nanjing 211816 China

**Keywords:** 2D materials, conductive filament, electrochemical metallization memristor, in situ transmission electron microscopy, metal sulfide

## Abstract

2D materials have drawn widespread attention as promising candidates for electrochemical metallization (ECM) memristors. However, some critical questions related to the resistance switching (RS) behaviors of 2D material‐based ECM memristors, such as the pathways of conductive filaments (CFs) growth/dissolution, the chemical composition and crystal structure of the CFs, remain largely unexplored. Herein, in situ transmission electron microscopy is employed to investigate the evolution of CFs in Ag (or Cu)/MoS_2_/W ECM memristors. Contrary to the traditional ECM theory, the CFs are found to grow from the anode to the cathode and dissolve from the cathode to the anode. Notably, Ag CFs with different crystal structures and metallic sulfide‐type CFs are observed in the memristors. These results provide deeper insights into the RS mechanism in 2D material‐based ECM memristors and facilitate the optimization of memristive devices.

## Graphical abstract

The evolution dynamics of conductive filaments (CFs) in 2D material‐based electrochemical metallization (ECM) memristor are studied under electric fields using in situ transmission electron microscopy. The growth/dissolution pathway is found to be contrary to traditional ECM theory. Ag CFs with face‐centered cubic (fcc) structure and hexagonal (4H) crystal structure, as well as metallic sulfide‐type CFs, are observed at the nanoscale.

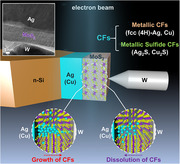

## 1 Introduction

With the rise of big data and artificial intelligence, the conventional von Neumann architecture is facing severe challenges in achieving highly efficient data processing and storage due to its discrete nature of computing and storage. It is necessary to develop novel storage and computational architectures to overcome the von Neumann bottleneck. Memristors, with a simple metal/insulator/metal (MIM) structure, are considered as promising candidates that exhibit great potential for in‐memory computing and large‐scale neuromorphic network applications.^[^ ^1^, ^2^, ^3^, ^4^, ^5^ ^]^ Among them, the electrochemical metallization (ECM) memristor is particularly representative.^[^ ^6^, ^7^, ^8^, ^9^ ^]^ The working mechanism of the ECM memristors is based on the formation and dissolution of conductive filaments (CFs) under the electric field, enabling the reversible resistance switching (RS) of the device between a high resistance state (HRS) and a low resistance state (LRS), namely the “SET” and “RESET” processes.^[^ ^10^, ^11^ ^]^ Currently, ECM memristors are mainly based on traditional bulk materials and have achieved some success.^[^ ^12^, ^13^, ^14^, ^15^ ^]^ However, their 3D structure inevitably constrains high‐density integration. Due to atomic thickness and unique physical properties, 2D materials are attracting significant attention, providing promising building blocks for ECM memristors.^[^ ^16^, ^17^, ^18^, ^19^ ^]^ The MIM configuration of ECM devices can be fabricated by incorporating 2D materials in a vertical or lateral stack between electrodes. Compared to lateral devices, vertical ECM memristors based on 2D materials offer superior integration capabilities, fast switching speeds, high on/off ratios, low SET/RESET voltages, good retention, and low energy consumption.^[^ ^20^, ^21^, ^22^, ^23^, ^24^ ^]^

Despite the fascinating progress, the underlying mechanism of the RS characteristics in 2D material‐based ECM memristors has yet to be fully elucidated, which could impede the optimization of these devices. In memristors based on monolayer 2D materials (such as MoS_2_ and h‐BN), the RS is attributed to the adsorption and desorption of metal ions at the point defects of the materials.^[^ ^25^, ^26^, ^27^ ^]^ In the case of multilayer 2D material‐based devices, the formation and dissolution of metallic CFs within the 2D materials, similar to traditional oxide bulk material‐based ECM memristors, is believed to be the main cause of the RS behaviors.^[^ ^28^, ^29^, ^30^ ^]^ Amorphous oxide bulk materials have ample interstitial spaces, while most 2D materials are compact layered crystalline in nature without a natural channel for metal ion migration across/through the multilayer structure. Therefore, the dynamics of CFs in memristors based on 2D materials may be different from those in bulk material‐based memristors. On the one hand, structural defects are required to form ion migration channels. On the other hand, chemical reactions may occur between metal ions from the electrodes and ions from the 2D materials’ lattices. Although early attempts, primarily based on cross‐sectional transmission electron microscopy (TEM) studies of pre‐switched devices and in situ conductive atomic force microscopy characterization, have been made to reveal the evolution mechanisms of CFs within 2D material‐based memristors,^[^ ^31^, ^32^, ^33^ ^]^ these provide only limited information about the CFs. The details about the migration of metal ions through the 2D multilayers, the formation and rupture process of CFs, and the chemical composition or crystalline structure of CFs during the RS operation remain largely unexplored.

In this work, the real‐time dynamic evolution of the CFs in 2D MoS_2_‐based memristors is systematically elucidated at the nanoscale through in situ TEM observations. Several fundamental issues related to the RS effect are revealed, including the pathway of CF growth/dissolution, the chemical composition, and the crystal structure of the CFs. Contrary to the traditional ECM theory, the CFs are found to grow from the anode to the cathode and dissolve from the cathode back to the anode. Impressively, Ag CFs with a hexagonal crystal structure (4H‐Ag) are first observed in the Ag/MoS_2_/W memristors. Furthermore, besides the commonly reported pure metal (e.g., Ag or Cu) filaments, metallic sulfide‐type CFs are also observed in the MoS_2_‐based ECM memristors. This may mainly result from the chemical reaction of metal ions with the free sulfur ions within the MoS_2_ lattice. These findings provide valuable insights into the RS mechanism in 2D material‐based ECM memristors and may facilitate the optimization of related memristive devices.

## 2 Results and Discussion

Here, chemical vapor deposition (CVD)‐synthesized MoS_2_ films with a thickness ranging from several nanometers to tens of nanometers were used as the RS medium. The films were characterized using Raman spectroscopy, X‐ray photoelectron spectroscopy (XPS), and TEM, confirming their relatively good quality and the presence of local atom vacancies (Figures S1 and S2, Supporting Information). For the fabrication of the ECM vertical memristor, the MoS_2_ films were transferred onto an Ag (or Cu) bottom electrode deposited on conductive n‐doped silicon substrates. Then, Au patterns used as the protection layers were transferred onto the top of the MoS_2_ film to form vertical Ag (or Cu)/MoS_2_/Au structures (Figure S3, Supporting Information). This process avoids lithography, which helps to minimize the damage to the MoS_2_ during device fabrication. Cross‐sectional specimens for in situ TEM studies were acquired using a focused ion beam (FIB) system. Both the thickness and lateral size of the sample were strictly controlled through the FIB milling process to enable high‐resolution observation of structural changes in the memristors.

**Figure 1:**
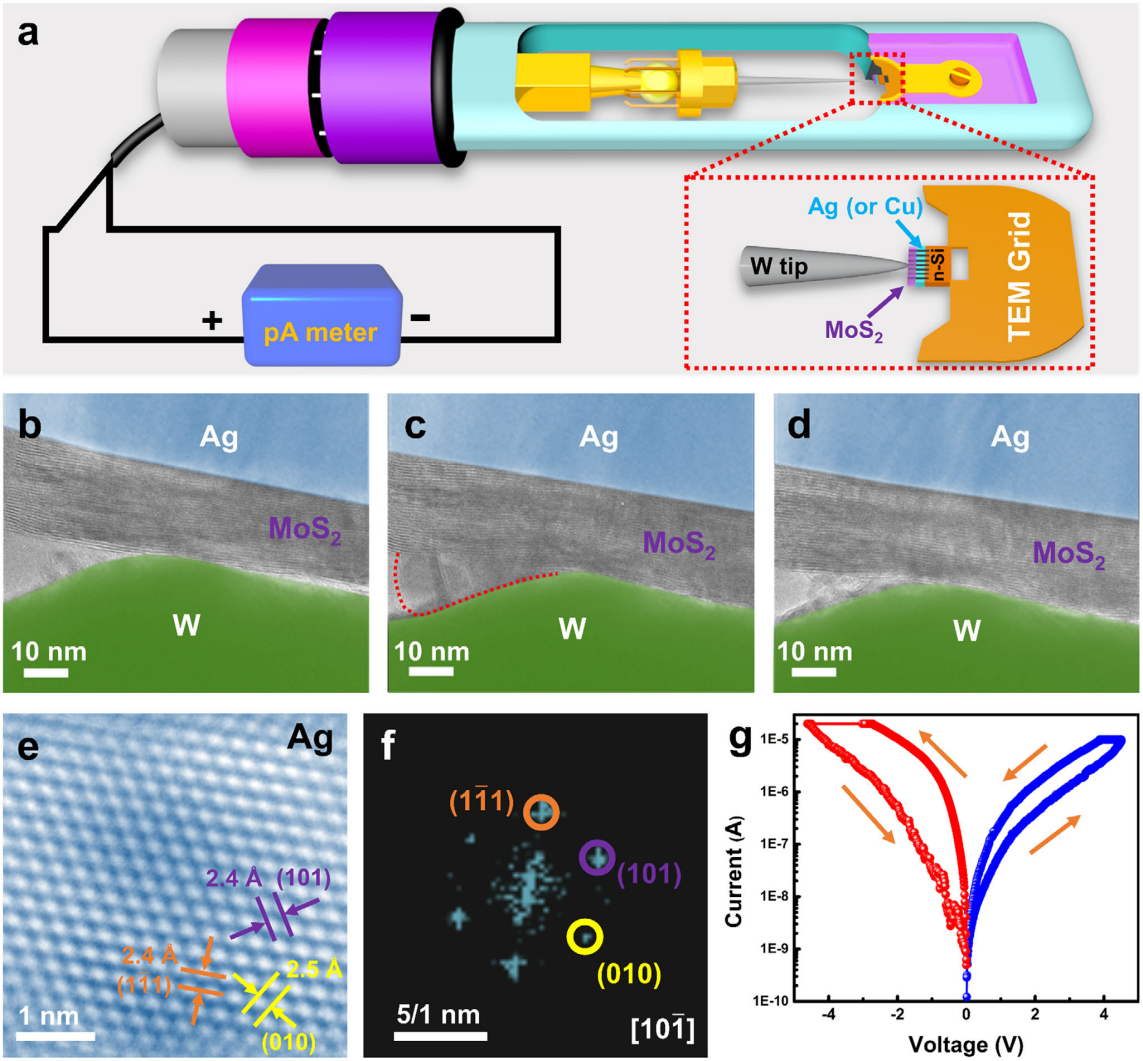
Construction of Ag (or Cu)/MoS_2_/W vertical memristors and in situ electrical measurement. a) Schematic illustration of the construction of the memristors. b–d) Cross‐sectional TEM images of (b) a fresh Ag/MoS_2_/W memristor, (c) the memristor in the LRS, and (d) the device after a RESET operation, respectively. The CF in (c) is highlighted by the red dotted line. e) HRTEM image of the CF region. f) The corresponding fast Fourier transform (FFT) pattern of (e). g) In situ *I–V* characteristic curve showing a bipolar switching behavior of the memristor. The voltage sweep direction is indicated by the arrows.

ECM memristors based on MoS_2_ films were constructed inside a Cs‐corrected TEM equipped with a TEM‐scanning tunneling microscopy (TEM‐STM) holder. As schematically illustrated in **Figure** **1**a, the cross‐sectional sample welded onto a TEM grid was fixed on the right side, while a W tip acting as the inert electrode was mounted on a movable piezo manipulator on the left side. The W tip was then controlled to contact the sample and scrape off the Pt and Au protective layers covering the top of the MoS_2_ film, thereby enabling the subsequent directed attachment to the MoS_2_ layers to form an Ag (or Cu)/MoS_2_/W vertical structure (Figure S4, Supporting Information). This approach facilitates the direct monitoring of microstructural changes within a smaller (nanometer‐scale) region, as the W tip is several nanometers to tens of nanometers in size. During the electrical measurement, the voltage was applied to the TEM grid while the W tip was grounded.

Figure 1b shows the cross‐sectional image of a pristine Ag/MoS_2_/W memristor, which is typically in the high‐resistance OFF‐state. Figure 1c,d show the images of the same device after being switched to the ON‐state and subsequently reset to the OFF‐state, respectively. After several positive voltage sweeps, a CF (marked by the red dotted line) suddenly appeared (Figure 1c), indicating that the RS behavior originates from the formation of CF. Notably, the portion of the CF located within the MoS_2_ was not readily apparent due to the relatively greater thickness and lattice structure of MoS_2_, which hindered its clear imaging. Conversely, when a negative sweep voltage was applied, the device abruptly switched back to the HRS, accompanied by the disappearance of the CF (Figure 1d). High‐resolution TEM (HRTEM) image (Figure 1e) and its corresponding FFT pattern (Figure 1f) confirm that the CF is composed of crystalline Ag. Figure 1g shows the corresponding *I–V* curve, which exhibits a bipolar switching behavior. During the positive voltage sweep, the device current rapidly increased at a voltage of 4.5 V and saturated to a compliance current of 10 µA, indicating the fresh device transitioned from the HRS to the LRS. Subsequently, during the negative voltage sweep, the device abruptly switched back to the HRS.

**Figure 2:**
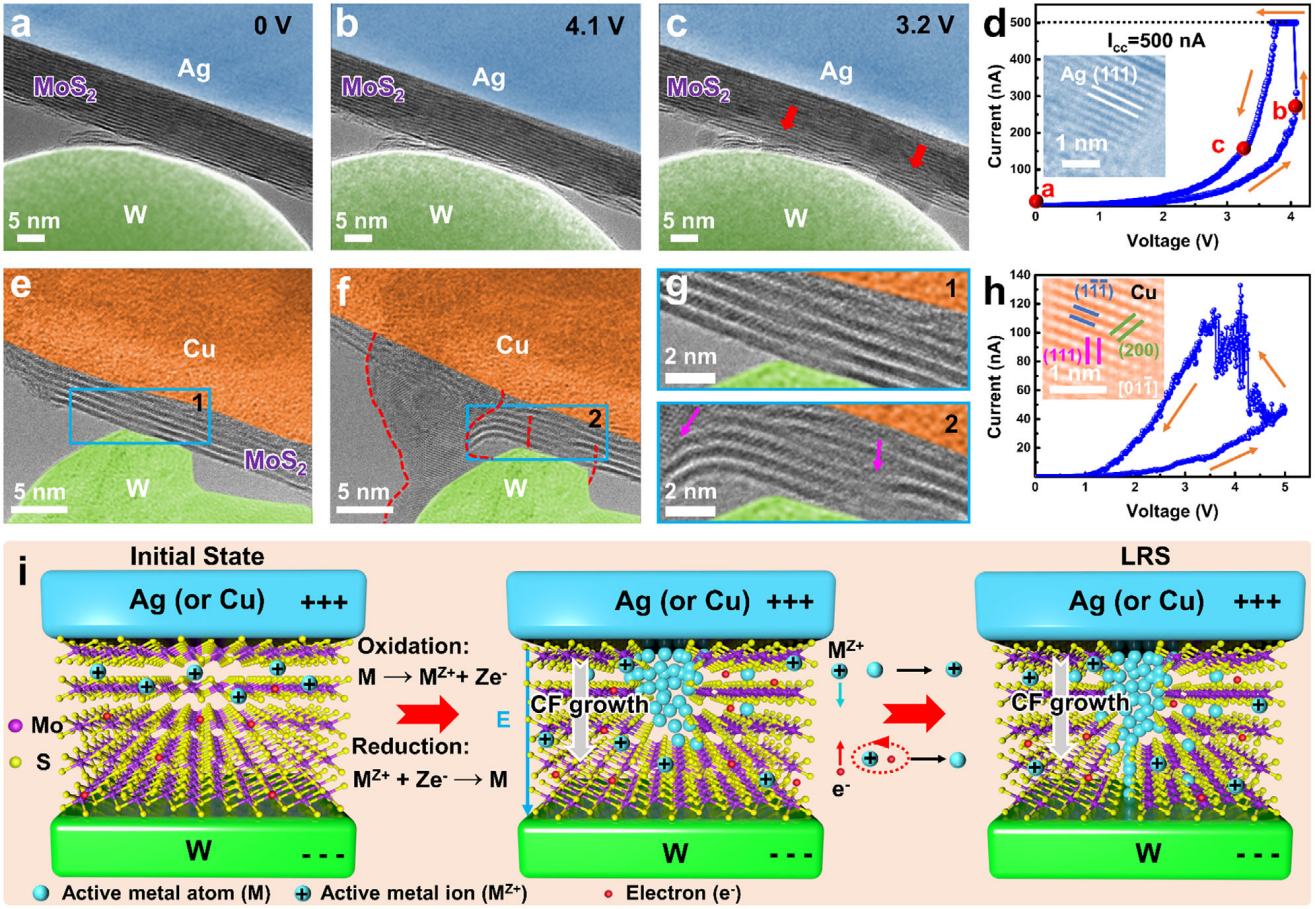
Dynamic growth of the CFs and the corresponding RS behavior. a–c) TEM images and corresponding d) *I‐* *V* characteristics of an Ag/MoS_2_/W memristor under the positive sweep voltage. The inset shows a HRTEM image of the CF. e,f) TEM images of a fresh Cu/MoS_2_/W memristor and the device in the LRS, respectively. g) Enlarged TEM images of the regions highlighted by blue rectangles in (e,f). h) *I*–*V* curve of the Cu/MoS_2_/W device under the positive voltage sweep. The inset shows a HRTEM image of the CF. i) Schematic illustrations depicting the mechanism of CF growth.

To further study the dynamic growth of the CFs, a positive sweep voltage was applied to an Ag/MoS_2_/W memristor. The growth of the CFs with voltage changes was recorded, and representative frames are shown in **Figure** **2**a–c. Upon the application of the positive sweep voltage, the MoS_2_ layer exhibits pronounced deformation, indicating the migration of Ag ions through the basal layer and the formation of migration channels. As the voltage reaches ≈4.1 V, the electrical current exhibits a significant surge and rapidly reaches the compliance current (I_cc_) of 500 nA (Figure 2d), demonstrating the complete CF formation, although it was not visibly apparent at that moment. Subsequently, as the voltage gradually drops to 3.2 V, the continued growth of CFs makes them clearly visible (indicated by the red arrows in Figure 2c). The corresponding HRTEM image (inset in Figure 2d) indicates that the composition of the CFs is Ag. Furthermore, the growth of CFs was also studied in a Cu/MoS_2_/W memristor (Figure 2e–h). Similarly, obvious CFs (highlighted by red dashed lines in Figure 2f) are observed in the device after the application of a positive voltage sweep, spanning across the MoS_2_ layers and bridging the Cu and W electrodes. Noticeable distortion of MoS_2_ layers is evident (indicated by arrows in Figure 2g), and the MoS_2_ lattice is disrupted, which facilitates the subsequent injection and migration of Cu ions, thereby leading to the nucleation and formation of CFs. The formation of CFs is responsible for the device switching from the HRS to the LRS (Figure 2h). HRTEM image in the inset of Figure 2h suggests the CFs are composed of Cu with a body‐centered cubic structure. Additionally, the observed downward bending of MoS_2_ layers suggests that CF growth proceeds from the anode toward the cathode, which is also consistent with the observation in other Cu/MoS_2_/W and Cu/MoS_2_/Pt devices (Figures S5 and S6, Supporting Information).

The CF growth from the anode toward the cathode deviates from the conventional ECM theoretical model, which predicts that metallic precipitates nucleate at the cathode and subsequently extend toward the anode. According to the conventional ECM mechanism, metallic atoms at the anode are oxidized into metal ions under a positive bias. These ions then migrate through the switching layer and are reduced at the inert cathode if they do not capture electrons during their transport through the RS medium. This behavior is well established for traditional solid‐state electrolytes such as GeS*_x_* and GeSe*_x_*,^[^ ^34^, ^35^ ^]^ which exhibit high solubility and mobility for specific metal ions. For instance, Ag filaments have been observed extending from the Au cathode toward the Ag anode in an Ag/As_2_S_3_: Ag/Au structure,^[^ ^36^ ^]^ and Cu filaments have been reported growing from the Pt‐Ir cathode toward the Cu anode in a Cu/Cu‐GeTe/Pt‐Ir device.^[^ ^37^ ^]^

In contrast, in the Ag (or Cu)/MoS_2_/W devices investigated in this study, the unique layered structure of 2D MoS_2_ is expected to exhibit substantially lower metal‐ion mobility than that of conventional solid‐state electrolytes, rendering the reduction of metal ions within the MoS_2_ film a plausible pathway. On this basis, the CF growth in MoS_2_‐based ECM memristors is described as shown in Figure 2i. Upon application of a positive voltage to the active electrode (Ag or Cu), metal atoms (M) are oxidized to form mobile metal ions (M^Z+^), which drift toward the cathode under the applied electric field. Due to their limited mobility in MoS_2_, metal ions are reduced to metallic atoms after only a short migration distance. Repeated oxidation‐reduction cycles promote the progressive accumulation of metal atoms at the anode/MoS_2_ interface, eventually leading to CF nucleation in localized regions. Once formed, the CF nucleus effectively extends the anode, creating a preferential pathway for ion migration toward the CF tip under the intensified local electric field.^[^ ^38^ ^]^ As the CF elongates, the layered MoS_2_ structure undergoes distortion and destruction, which reduces migration barriers and further facilitates ion transport. With continued growth, the CF eventually bridges the cathode, forming a highly conductive pathway through the MoS_2_ layers and switching the device to the LRS.

Considering that the device is exposed to electron beam irradiation during the dynamical observation, control experiments were conducted with the electron beam switched off to evaluate the influence of electron beam‐induced artifacts (Figure S7, Supporting Information). The results confirm that the electron beam irradiation has little effect on CF formation and that the electric field is the primary driver of CF growth.

**Figure 3:**
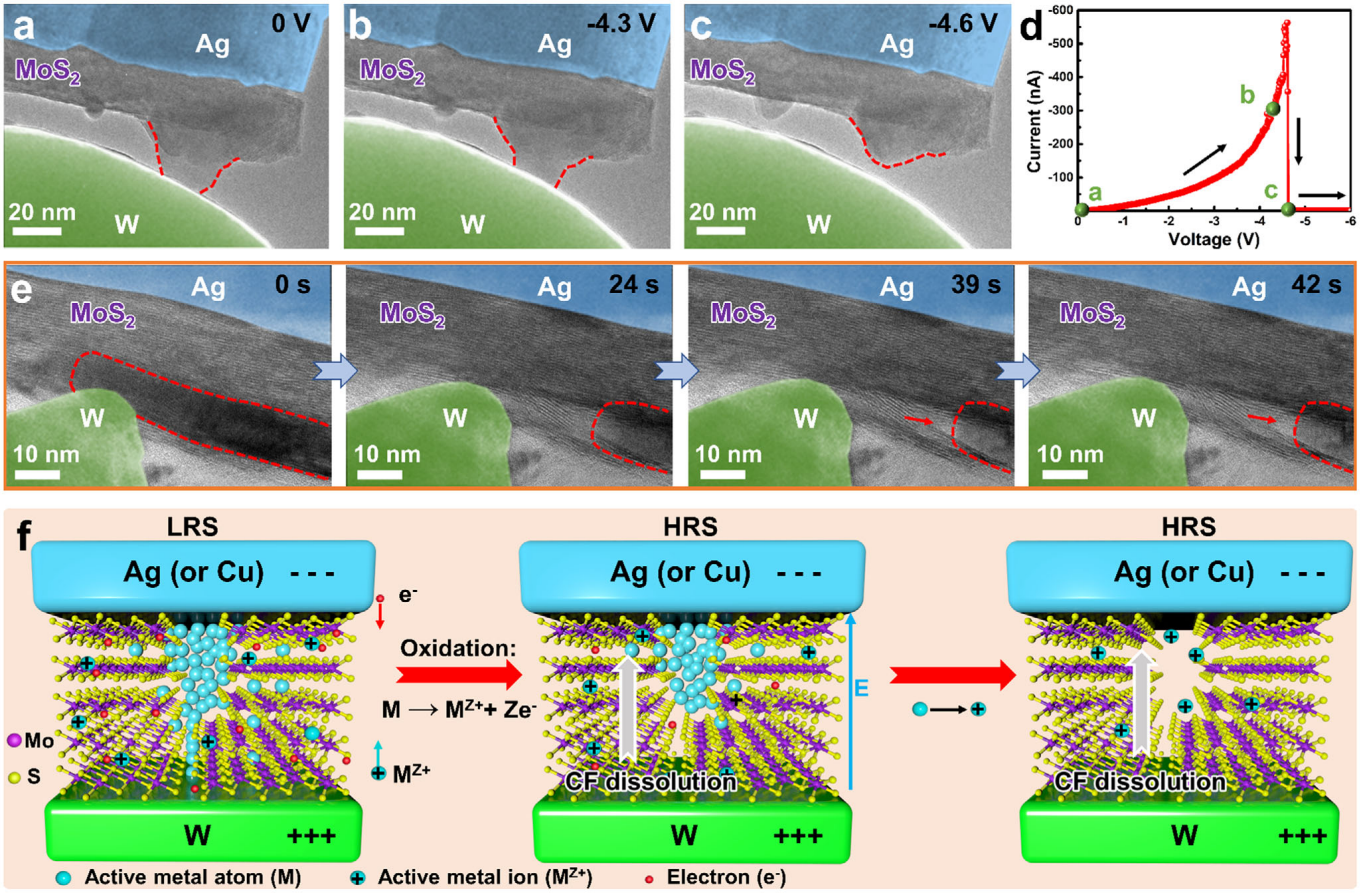
Dissolution of the CFs and the corresponding RS behaviors. a–c) TEM images of an Ag/MoS_2_/W memristor and corresponding d) *I–V* characteristics under the negative voltage sweep. e) Time‐sequence TEM images illustrating CF dissolution in another Ag/MoS_2_/W memristor. f) Schematic illustrations showing the mechanism of CF dissolution.

Besides, the dissolution of the CFs was also investigated in the Ag (or Cu)/MoS_2_/W memristors (**Figure** **3**; Figure S8, Supporting Information). The memristors were first switched to the LRS, after which negative sweep voltages were applied while recording the dissolution dynamics. Figure 3a–c presents CF dissolution in an Ag/MoS_2_/W memristor under a negative sweep voltage (0 V → −6 V). As the voltage increases, the tip of the CF connected to the W electrode thins and eventually ruptures, leaving some residuals and causing a drop in electrical current (Figure 3d). During CF dissolution, the structural deformation in MoS_2_ induced by CF formation can partially recover upon CF complete dissolution (Figure S9, Supporting Information). Nevertheless, in some cases, significant lattice damage caused by CF formation, coupled with incomplete CF dissolution, results in residues embedded within the MoS_2_, thereby maintaining relatively stable channels. A representative example is shown in Figure 3e, where a CF embedded in the MoS_2_ interlayer (outlined by red dashed lines) progressively dissolves and retracts from the W electrode under a negative sweep voltage. After 39 s, dissolution largely ceases, leaving a stabilized CF residue within the MoS_2_. This incomplete dissolution of CF preserves an enlarged interlayer spacing (indicated by red arrows), thereby sustaining a channel for subsequent CF reformation.

As discussed above, the growth direction of the CF is from the anode to the cathode. Consequently, the thinner part of the CF is located near the cathode (W electrode), which is obvious in Figure 3a. It is expected that the current density is highest at the narrowest point of the filament, where the local resistance is maximum. The combination of high current density and resistance produces intense localized Joule heating, facilitating oxidation and dissolution of the CF. As a result, the rupture of the CF occurs at the CF/W electrode interface, and dissolution proceeds from the W electrode toward the Ag (or Cu) electrode (Figure 3f). During the dissolution process, some residuals of the CF may remain due to the partial dissolution. Under multiple RESET operations or one RESET process with a longer duration, the CF may completely dissolve. These residuals could influence the RS performance. On the one hand, some smaller residuals can serve as nucleation sites for the reformation of complete CFs during the subsequent SET process. On the other hand, however, more robust residuals may hinder the successful RESET process, causing the device to maintain the LRS. Therefore, the control of the size of the initial CF is critical during the CF formation process, and appropriate voltages and compliance currents should be applied.

**Figure 4:**
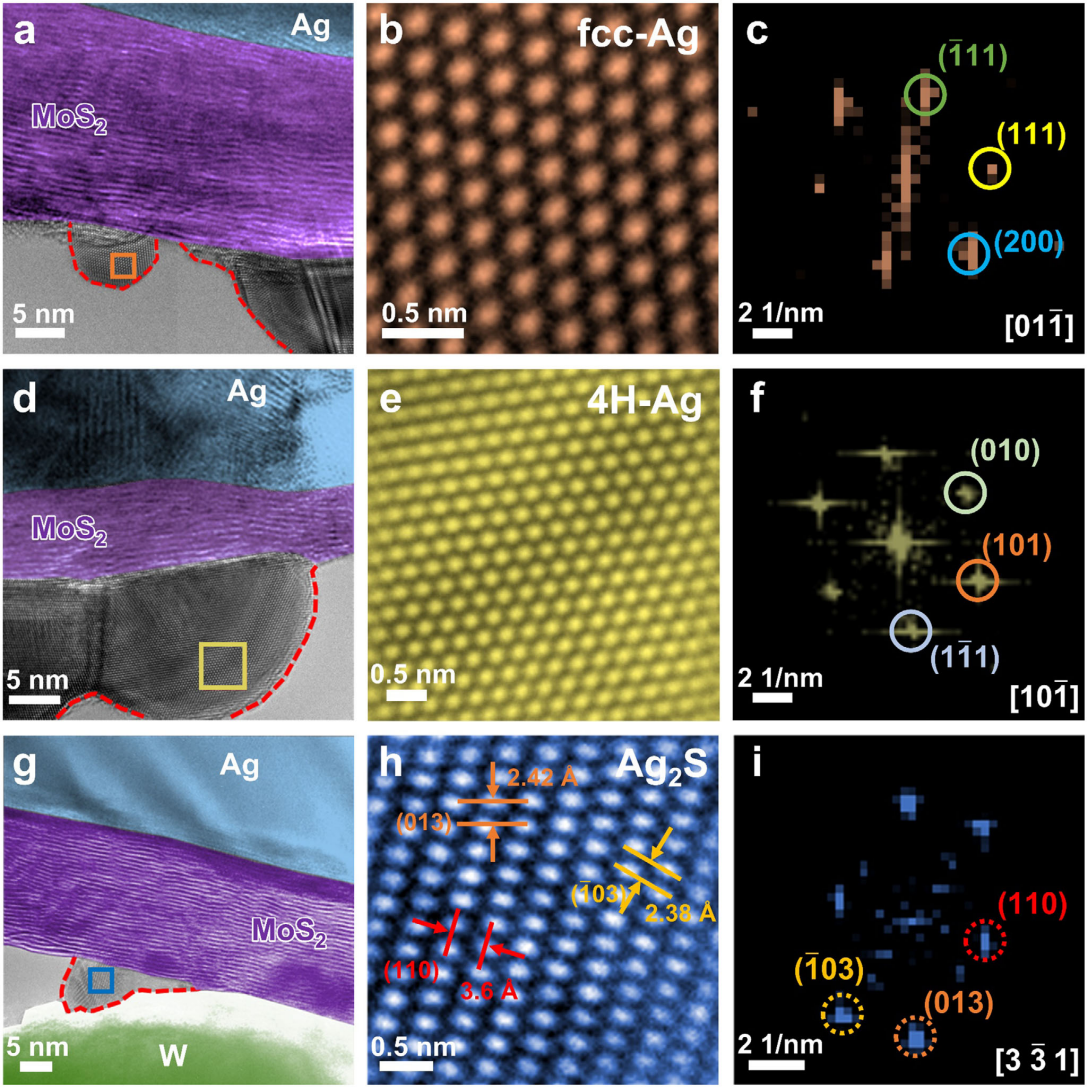
Metal CFs and metal sulfide CFs in the Ag/MoS_2_/W memristors. a,d,g) TEM images of Ag/MoS_2_/W memristors in the LRS. b,e,h) HRTEM images of the regions framed by the orange, yellow, and blue boxes in (a), (d), and (g), respectively. c,f,i) Corresponding FFT patterns of (b), (e), and (h), respectively.

In addition to the growth/dissolution dynamics of the CFs that can influence memristor performance, the chemical composition and crystalline structure of the CFs could also be crucial factors affecting the RS performance, as they typically determine the electrical resistance states of the memristors. Therefore, the consistency of the chemical composition and crystalline structure usually plays an important role in the uniformity of the device performance. In general, the CFs in ECM memristors are usually composed of pure active metal (such as Ag or Cu),^[^ ^9^, ^10^, ^11^, ^12^ ^]^ which is also confirmed in our in situ experiments. Moreover, in the ECM memristors with Ag as the active metal electrode, Ag with a face‐centered cubic (fcc) structure is comprehensively accepted as the composition of the CFs,^[^ ^6^, ^8^, ^30^ ^]^ which is commonly observed in our experiments (**Figure** **4**a–c; Figure S10, Supporting Information). Figure 4a presents the TEM image of a typical Ag/MoS_2_/W memristor that has been switched to the LRS, where the CFs are marked by red dashed lines. The corresponding HRTEM image and FFT pattern (Figure 4b,c) of the orange rectangle region confirm that the CF is composed of fcc‐Ag. Interestingly, in some cases (for example, Figure 4d–f), the Ag CFs exhibit a hexagonal crystal structure (4H‐Ag). These phenomena indicate that Ag atoms may crystallize into different crystal structures (including fcc‐Ag and 4H‐Ag) during the Ag CFs growth, which should be given much attention, as there are significant differences in the physical properties between fcc‐Ag and 4H‐Ag. Theoretical calculations suggest that 4H‐Ag exhibits ≈130 times higher in‐plane resistivity compared to the common fcc‐Ag.^[^ ^39^ ^]^ Therefore, the existence of Ag CFs with different crystal structures may lead to obvious resistance state differences, thereby affecting the uniformity of the device performance. The formation of 4H‐Ag CFs may be attributed to both the crystalline structure of the Ag electrode and the template effect of the MoS_2_ layer. It is highly probable that the Ag electrode prepared via electron beam evaporation or thermal evaporation contains 4H‐Ag components; a similar phenomenon has been previously reported in the preparation of Ag film by magnetron sputtering,^[^ ^40^ ^]^ which can serve as an epitaxial template for the formation of 4H‐Ag CFs. Additionally, the hexagonal lattice of MoS_2_ can also act as an epitaxial template for Ag deposition, favoring the formation of 4H‐Ag over fcc‐Ag due to minimal lattice mismatch. Since 4H‐Ag is metastable, it is expected to transform into fcc‐Ag after multiple electrical cycles, primarily driven by Joule heating.

Impressively, in addition to the conventional pure metallic CFs, metallic sulfide‐type CFs were also observed in our experiments. Figure 4g–i shows a Ag_2_S CF in an Ag/MoS_2_/W memristor. The HRTEM image (Figure 4h) of the CF region framed by a blue rectangle (Figure 4g) reveals typical lattice spacings of 3.6, 2.38, and 2.42 Å, which can be indexed as the (110), ($\bar{1}03$), and (013) planes of monoclinic Ag_2_S along the [$3\bar{3}1$] zone axis, confirming that the formed CF is Ag_2_S. Moreover, another example of an Ag_2_S CF is shown in Figure S11 (Supporting Information). Similarly, a CF composed of Cu_2_S was also found in a Cu/MoS_2_/W memristor (Figure S12, Supporting Information). These observations reveal that metallic sulfide‐type CFs could form in the MoS_2_‐based memristors, which has not been reported before. Moreover, since both Ag and Cu can form metal sulfides with varying stoichiometric ratios (such as Ag_1.96_S and Cu_9_S_5_), different metal sulfides (M*_x_* S_y_) may form depending on the specific situation. A previous study revealed that a Ti*_x_* S*_y_* layer forms at the Ti/MoS_2_ interface in Ti/MoS_2_/Pt memristors through XPS characterization. However, the Ti*_x_* S*_y_* layer only functions as a sulfur ion reservoir, while CFs consisting of sulfur vacancies lead to the RS phenomenon.^[^ ^41^ ^]^ In contrast, metal sulfide CFs were directly observed in our cases through in situ TEM, and these CFs directly determined the RS phenomenon, rather than relying on sulfur vacancies to form the CFs. It should be noted that although the exact composition of the internal CF region of MoS_2_ cannot be determined based on the external CF portion, this at least suggests that the metal sulfides participate in the overall composition of the CF and directly influence the RS behaviors.

**Figure 5:**
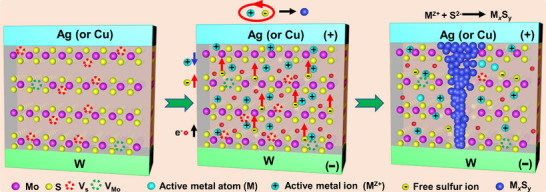
Schematic illustration of the possible formation mechanism of metal sulfide (M*_x_* S_y_) CFs through direct chemical reactions between metal ions and free sulfur ions.

The observed formation of metal sulfides suggests that a chemical reaction between migrating metal ions and sulfur ions may occur during CF growth. Specifically, one possible mechanism involves the direct chemical reaction of active metal ions with mobile free sulfur ions within the MoS_2_ matrix (**Figure** **5**). Crucially, CVD‐synthesized MoS_2_ film inherently possesses a high density of sulfur vacancies (V_s_), which serve as reservoirs for ionic sulfur species and as preferential sites for lattice distortion under an electric field. During CF formation, the interplay between the migrating metal ions and the MoS_2_ lattice, coupled with localized volume expansion, promotes the generation of additional sulfur vacancies via S─Mo bond rupture, thereby increasing the concentration of mobile free sulfur ions. Under the applied electric field, these liberated sulfur ions migrate counter to the electric field direction, while active metal ions from the anode drift toward the cathode. Upon encountering each other, the oppositely migrating ions undergo electrochemical reactions (M^z^⁺ + S^2−^ → M*_x_* S*_y_*), leading to the nucleation of metal sulfide clusters. Progressive coalescence of these clusters ultimately results in the formation of a metal sulfide CF extending to the cathode. Once the CF connects to the cathode, a notable decrease in the overall electrical resistance is observed.

Additionally, there may be another possibility, namely migrating metal ions could occupy molybdenum vacancies (V_Mo_) within the MoS_2_ lattice and subsequently form chemical bonds with adjacent sulfur ions, thereby leading to the formation of metallic sulfide CFs (Figures S13 and S14, Supporting Information). However, this pathway would necessitate the generation of a substantial population of molybdenum vacancies. Considering the relatively high formation energy barrier associated with such vacancies,^[^ ^42^ ^]^ this mechanism is therefore unlikely to represent the dominant route for metallic sulfide CF formation. It is noteworthy that during CF growth, the conventional ECM process may occur concurrently, potentially incorporating pure metal (Ag/Cu) into the final composition of the CFs.

The discovery of metal sulfide CFs has expanded the diversity of available CFs. On the one hand, metal sulfides are typically semiconductors with electrical conductivity significantly different from that of pure metals, which may affect the performance uniformity of memristors. On the other hand, the existence of metal sulfide CFs could introduce additional electrical resistance states, thereby facilitating multi‐state storage applications based on 2D materials. Given these potential implications, a rational device design should be considered to harness the benefits while mitigating the potential drawbacks. This may involve optimizing the material composition and device structure to ensure consistent CF formation and reliable RS behavior. For example, strategies include preparing high‐quality 2D materials with fewer vacancies, inserting a nanoscale Al_2_O_3_ layer between the active metal electrode and MoS_2_ to prevent direct reactions between metal ions and sulfur ions, or applying nanosecond electric pulses (<100 ns) to minimize Joule heating and avoid long‐range sulfur ion migration. Additionally, further investigation into the underlying mechanisms and the factors influencing the formation of metal sulfide CFs could provide valuable insights for device engineering and performance optimization.

## 3 Conclusion

In summary, the real‐time dynamic evolution of CFs in 2D MoS_2_‐based vertical ECM memristors was systematically investigated at the nanometer scale through in situ TEM observations. Contrary to the traditional ECM theory, the CFs in the MoS_2_‐based memristors were found to grow from the anode toward the cathode, and dissolve from the cathode back to the anode. This growth/dissolution mode is attributed to the low metal ion mobility across the layers of the 2D MoS_2_. Intriguingly, Ag CFs with both fcc and hexagonal crystal structures were found in the Ag/MoS_2_/W memristors. Furthermore, metallic sulfide‐type CFs were also observed in the memristors, resulting from the chemical reaction of metal ions with the free sulfur ions. The discovery of metallic sulfide‐type CFs could facilitate multi‐state storage applications based on 2D materials. These findings provide fundamental insights into the RS mechanism in 2D material‐based ECM memristors and could promote the further optimization of memristive devices based on 2D materials.

## 4 Experimental Section

### Preparation of the Ag (or Cu)/MoS_2_/Au structures

First, a 30 nm Ag (or Cu) electrode with a 4 nm Ti (or Cr) adhesion layer was deposited on *n*‐type heavily doped conductive silicon substrate by electron beam evaporation or thermal evaporation. Second, MoS_2_ films purchased from Shenzhen SixCarbon Technology Co., Ltd, were transferred onto the Ag (or Cu) electrode through a surface‐energy‐assisted transfer process.^[^ ^43^, ^44^ ^]^ Finally, a 30 nm Au pattern as the protection layer was transferred on top of the MoS_2_ film to form Ag (or Cu)/MoS_2_/Au structures (see more details in Supporting Information).

### In Situ TEM Experiments

The cross‐sectional TEM specimens were prepared using a dual‐beam FIB system (Thermo Scientific Helios 5CX). During the fabrication process, the samples were thinned down by ion beam milling with an accelerating voltage of 30 kV and low beam currents, followed by two steps of fine polishing with low‐energy Ga ions milling (5 kV, 41 pA and 2 kV, 39 pA, respectively). In situ TEM experiments were conducted in a Cs‐corrected TEM (FEI Titan 80–300) operated at 300 kV. The sample was fixed on one side of the TEM‐STM holder (Zeeptools PicoFemto). A W tip, serving as the inert electrode, was controlled to scratch the top Pt and Au protective layers of the sample to subsequently contact with the MoS_2_ film, forming an Ag (or Cu)/MoS_2_/W vertical memristor structure. Electrical measurements were performed by applying bias to the TEM grid with a Keithley 6430 analyzer while the W tip was grounded.

## Conflict of Interest

The authors declare no conflict of interest.

## Supporting information



Supporting Information

## Data Availability

The data that support the findings of this study are available from the corresponding author upon reasonable request.

## References

[1] D. B. Strukov , G. S. Snider , D. R. Stewart , R. S. Williams , Nature 2008, 453, 80. 18451858 10.1038/nature06932

[2] M. Lanza , A. Sebastian , W. D. Lu , M. L. Gallo , M.‐F. Chang , D. Akinwande , F. M. Puglisi , H. N. Alshareef , M. Liu , J. B. Roldan , Science 2022, 376, abj9979. 10.1126/science.abj9979 35653464

[3] W. Zhang , B. Gao , J. Tang , P. Yao , S. Yu , M.‐F. Chang , H.‐J. Yoo , H. Qian , H. Wu , Nat. Electron. 2020, 3, 371.

[4] X. Li , J. Tang , Q. Zhang , B. Gao , J. J. Yang , S. Song , W. Wu , W. Zhang , P. Yao , N. Deng , L. Deng , Y. Xie , H. Qian , H. Wu , Nat. Nanotechnol. 2020, 15, 776. 32601451 10.1038/s41565-020-0722-5

[5] F. Zhou , Z. Zhou , J. Chen , T. H. Choy , J. Wang , N. Zhang , Z. Lin , S. Yu , J. Kang , H.‐S. P. Wong , Y. Chai , Nat. Nanotechnol. 2019, 14, 776. 31308498 10.1038/s41565-019-0501-3

[6] Y. Yang , P. Gao , S. Gaba , T. Chang , X. Pan , W. Lu , Nat. Commun. 2012, 3, 732. 22415823 10.1038/ncomms1737

[7] H. Yeon , P. Lin , C. Choi , S. H. Tan , Y. Park , D. Lee , J. Lee , F. Xu , B. Gao , H. Wu , H. Qian , Y. Nie , S. Kim , J. Kim , Nat. Nanotechnol. 2020, 15, 574. 32514010 10.1038/s41565-020-0694-5

[8] C. Li , T. Xu , R. Pan , S. Bao , K. Yin , J. Shen , Y. Zhu , S. Hou , L. Sun , ACS Nano 2024, 18, 32196. 39499731 10.1021/acsnano.4c11598

[9] S. A. Chekol , S. Menzel , R. W. Ahmad , R. Waser , S. Hoffmann‐Eifert , Adv. Funct. Mater. 2022, 32, 2111242.

[10] R. Waser , M. Aono , Nat. Mater. 2007, 6, 833. 17972938 10.1038/nmat2023

[11] A. Roy , P.‐R. Cha , J. Appl. Phys. 2020, 128, 205102.

[12] Z. Wang , M. Rao , R. Midya , S. Joshi , H. Jiang , P. Lin , W. Song , S. Asapu , Y. Zhuo , C. Li , H. Wu , Q. Xia , J. J. Yang , Adv. Funct. Mater. 2018, 28, 1704862.

[13] A. Korneluk , T. Stefaniuk , Adv. Mater. 2025, 37, 2411186. 39564711 10.1002/adma.202411186 PMC11756036

[14] J. H. Yoon , J. Zhang , P. Lin , N. Upadhyay , P. Yan , Y. Liu , Q. Xia , J. J. Yang , Adv. Mater. 2020, 32, 1904599. 10.1002/adma.201904599 31984587

[15] K. S. Woo , H. Park , N. Ghenzi , A. A. Talin , T. Jeong , J.‐H. Choi , S. Oh , Y. H. Jang , J. Han , R. S. Williams , S. Kumar , C. S. Hwang , ACS Nano 2024, 18, 17007. 38952324 10.1021/acsnano.4c03238

[16] M. Li , H. Liu , R. Zhao , F.‐S. Yang , M. Chen , Y. Zhuo , C. Zhou , H. Wang , Y.‐F. Lin , J. J. Yang , Nat. Electron. 2023, 6, 491.

[17] K. C. Kwon , J. H. Baek , K. Hong , S. Y. Kim , H. W. Jang , Nano‐Micro Lett. 2022, 14, 58. 10.1007/s40820-021-00784-3 PMC8818077 35122527

[18] L. Yin , R. Cheng , Y. Wen , C. Liu , J. He , Adv. Mater. 2021, 33, 2007081. 10.1002/adma.202007081 34105195

[19] H. Zhou , S. Li , K.‐W. Ang , Y.‐W. Zhang , Nano‐Micro Lett. 2024, 16, 121. 10.1007/s40820-024-01335-2 PMC10876512 38372805

[20] X. F. Lu , Y. Zhang , N. Wang , S. Luo , K. Peng , L. Wang , H. Chen , W. Gao , X. H. Chen , Y. Bao , G. Liang , K. P. Loh , Nano Lett. 2021, 21, 8800. 34644096 10.1021/acs.nanolett.1c03169

[21] S. Chen , M. R. Mahmoodi , Y. Shi , C. Mahata , B. Yuan , X. Liang , C. Wen , F. Hui , D. Akinwande , D. B. Strukov , M. Lanza , Nat. Electron. 2020, 3, 638.

[22] P. Lei , H. Duan , L. Qin , X. Wei , R. Tao , Z. Wang , F. Guo , M. Song , W. Jie , J. Hao , Adv. Funct. Mater. 2022, 32, 2201276.

[23] Y. Li , L. Loh , S. Li , L. Chen , B. Li , M. Bosman , K.‐W. Ang , Nat. Electron. 2021, 4, 348.

[24] J. Tang , C. He , J. Tang , K. Yue , Q. Zhang , Y. Liu , Q. Wang , S. Wang , N. Li , C. Shen , Y. Zhao , J. Liu , J. Yuan , Z. Wei , J. Li , K. Watanabe , T. Taniguchi , D. Shang , S. Wang , W. Yang , R. Yang , D. Shi , G. Zhang , Adv. Funct. Mater. 2021, 31, 2011083.

[25] S. M. Hus , R. Ge , P.‐A. Chen , L. Liang , G. E. Donnelly , W. Ko , F. Huang , M.‐H. Chiang , A.‐P. Li , D. Akinwande , Nat. Nanotechnol. 2021, 16, 58. 33169008 10.1038/s41565-020-00789-w

[26] X. Wu , R. Ge , P.‐A. Chen , H. Chou , Z. Zhang , Y. Zhang , S. Banerjee , M.‐H. Chiang , J. C. Lee , D. Akinwande , Adv. Mater. 2019, 31, 1806790. 10.1002/adma.201806790 30773734

[27] S. Mitra , S. Mahapatra , npj 2D Mater. Appl. 2024, 8, 26.

[28] Y. Shi , X. Liang , B. Yuan , V. Chen , H. Li , F. Hui , Z. Yu , F. Yuan , E. Pop , H.‐S. P. Wong , M. Lanza , Nat. Electron. 2018, 1, 458.

[29] R. Xu , H. Jang , M.‐H. Lee , D. Amanov , Y. Cho , H. Kim , S. Park , H.‐J. Shin , D. Ham , Nano Lett. 2019, 19, 2411. 30896171 10.1021/acs.nanolett.8b05140

[30] K. Qian , R. Y. Tay , V. C. Nguyen , J. Wang , G. Cai , T. Chen , E. H. T. Teo , P. S. Lee , Adv. Funct. Mater. 2016, 26, 2176.

[31] W. Ahn , H. B. Jeong , J. Oh , W. Hong , J.‐H. Cha , H. Y. Jeong , S.‐Y. Choi , Small 2023, 19, 2300223. 10.1002/smll.202300223 37093184

[32] M. J. Lee , S.‐H. Kim , S. Lee , C. Yoon , K.‐A. Min , H. Choi , S. Hong , S. Lee , J.‐G. Park , J.‐P. Ahn , B. H. Park , NPG Asia Mater 2020, 12, 82.

[33] R. D. Nikam , H. Hwang , Adv. Funct. Mater. 2022, 32, 2201749.

[34] R. Waser , R. Dittmann , G. Staikov , K. Szot , Adv. Mater. 2009, 21, 2632. 36751064 10.1002/adma.200900375

[35] M. N. Kozicki , M. Park , M. Mitkova , IEEE Trans. Nanotechnol. 2005, 4, 331.

[36] Y. Hirose , H. Hirose , J. Appl. Phys. 1976, 47, 2767.

[37] S.‐J. Choi , G.‐S. Park , K.‐H. Kim , S. Cho , W.‐Y. Yang , X.‐S. Li , J.‐H. Moon , K.‐J. Lee , K. Kim , Adv. Mater. 2011, 23, 3272. 21671452 10.1002/adma.201100507

[38] Q. Liu , J. Sun , H. Lv , S. Long , K. Yin , N. Wan , Y. Li , L. Sun , M. Liu , Adv. Mater. 2012, 24, 1844. 22407902 10.1002/adma.201104104

[39] I. Chakraborty , S. N. Shirodkar , S. Gohil , U. V. Waghmare , P. Ayyub , J. Phys.: Condens. Matter 2014, 26, 025402. 24305516 10.1088/0953-8984/26/2/025402

[40] P. Taneja , R. Banerjee , P. Ayyub , G. K. Dey , Phys. Rev. B 2001, 64, 033405.

[41] L. Liu , Y. Wang , W. Chen , S. Ren , J. Guo , X. Kang , X. Zhao , Appl. Surf. Sci. 2022, 605, 154698.

[42] D. Liu , Y. Guo , L. Fang , J. Robertson , Appl. Phys. Lett. 2013, 103, 183113.

[43] A. Gurarslan , Y. Yu , L. Su , Y. Yu , F. Suarez , S. Yao , Y. Zhu , M. Ozturk , Y. Zhang , L. Cao , ACS Nano 2014, 8, 11522. 25347296 10.1021/nn5057673

[44] S. M. Shinde , T. Das , A. T. Hoang , B. K. Sharma , X. Chen , J.‐H. Ahn , Adv. Funct. Mater. 2018, 28, 1706231.

