# Supplementary material for: Nanoscale Insights into the Dynamics of Conductive Filament Growth/Dissolution in 2D Material‐Based Memristors
Source: Adv Sci (Weinh). 2025 Oct 14;12(45):e09791. doi: 10.1002/advs.202509791 (PMC12677641; doi:10.1002/advs.202509791)
Supplement: Supplementary file 1 — Supporting Information [file ADVS-12-e09791-s001.docx]

Supporting Information

**Nanoscale Insights into the Dynamics of Conductive Filament Growth/Dissolution in 2D Material-Based Memristors**

*Chen Li, Rui Pan, Tao Xu*, Jiaxin shen, Yatong Zhu, Mingrui Zhou, Xiaohui Hu, Kuibo Yin, and Litao Sun**

C. Li, R. Pan, T. Xu, J. Shen, Y. Zhu, M. Zhou, K. Yin, L. Sun

SEU-FEI Nano-Pico Center, Key Laboratory of MEMS of Ministry of Education, Southeast University, Nanjing 210096, China

Email: [xt@seu.edu.cn](mailto:xt@seu.edu.cn); [slt@seu.edu.cn](mailto:slt@seu.edu.cn)

X. Hu

College of Materials Science and Engineering, Nanjing Tech University, Nanjing 211816, China

**S1. Raman and XPS characterization of the MoS_2_ film.**

Figure S1a presents the Raman spectrum of the MoS_2_ film, indicating two characteristic peaks centered at 382.94 cm^−1^ and 408.09 cm^−1^, which can be attributed to the $\text{E}_{\text{2g}}^{\text{1}}$ and $\text{A}_{\text{1g}}$ vibrational modes of MoS_2_, respectively. The chemical composition of the MoS_2_ films was characterized using XPS. The Mo 3d core level spectrum exhibits high peaks at 232.9 and 229.8 eV (Figure S1b), which can be attributed to Mo^4+^ 3d_3/2_ and Mo^4+^ 3d_5/2_, respectively. The S 2p core-level spectrum displays two peaks at 163.8 and 162.6 eV, corresponding to S^2−^ 2p_1/2_ and S^2−^ 2p_3/2_ (Figure S1c), respectively. Moreover, the atomic ratio of the MoS_2_ film was calculated using XPS peak data, and the atomic composition ratio for Mo and S was found to be 1:1.78 (Figure S1c), indicating the existence of sulfur vacancies in the CVD-synthesized MoS_2_ film. Defects in 2D materials can facilitate the formation and rupture of conductive filaments in ECM memristors based on 2D materials.


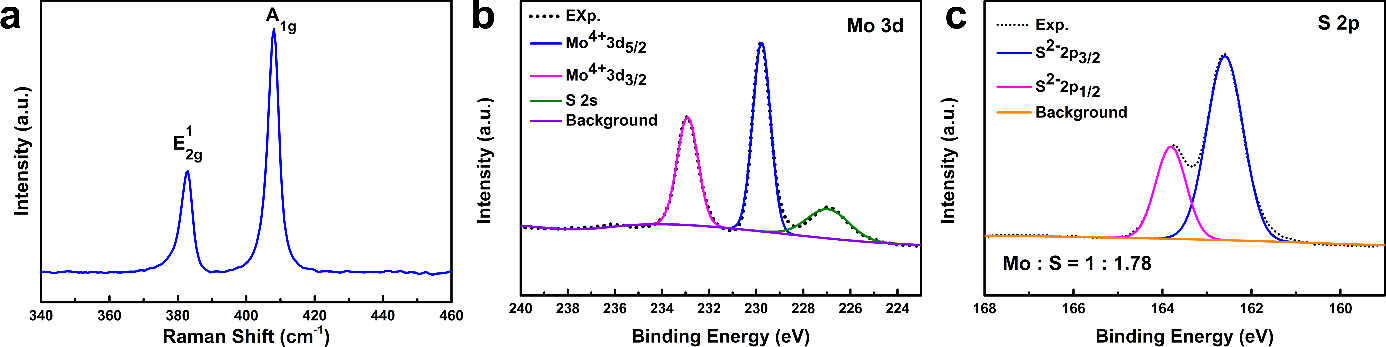


**Figure S1**. Raman and XPS characterization of the MoS_2_ film. (a) Raman spectrum of the MoS_2_ film, showing the characteristic $\text{E}_{\text{2g}}^{\text{1}}$ peak at 382.94 cm^−1^ and the $\text{A}_{\text{1g}}$ peak at 408.09 cm^−1^. (b, c) XPS spectra of Mo 3d and S 2p, respectively.

**S2. TEM characterization of the MoS_2_ film.**

The MoS_2_ film was characterized by TEM. Figure S2a shows the high-resolution TEM (HRTEM) image of the film, and the inset exhibits the selected area electron diffraction (SAED) pattern, indicating the polycrystalline nature of the sample. Figure S2b shows the lattice fringes with a distance of 2.73 Å, which can be indexed to the (100) plane of MoS_2_. Figure S2c shows an atomic-resolution annular dark-field scanning transmission electron microscopy (ADF-STEM) image, indicating the presence of multi-atom vacancies (as indicated by the arrows) in the film. The corresponding energy-dispersive X-ray spectroscopy (EDS) results (Figure S2d) confirm the elemental composition of Mo and S, and their uniform distribution throughout the film.

**
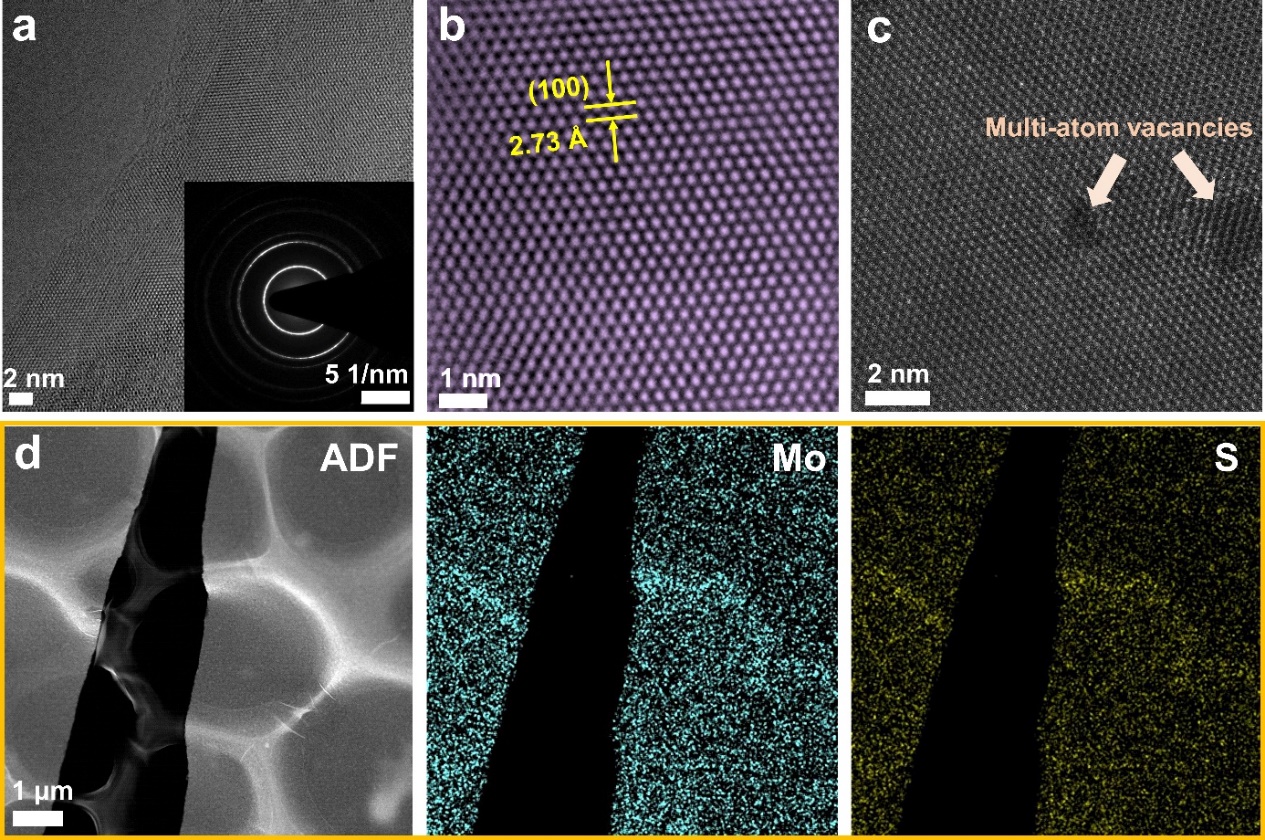
**

**Figure S2.** Characterization of the MoS_2_ film. (a) HRTEM image and SAED pattern (inset). (b) HRTEM image showing lattice fringes corresponding to the (100) plane of MoS_2_. (c) Atomic-resolution ADF-STEM image showing the presence of multi-atom vacancies (marked by arrows) in the MoS_2_ film. (d) Low-magnification ADF-STEM image and the corresponding EDS elemental mappings of Mo and S.

**S3. Experiment setup for the fabrication of Ag (or Cu)/MoS_2_/Au sandwich structures.**

Ag (or Cu)/MoS_2_/Au sandwich structures for in situ TEM experiments were fabricated as shown in Figure S3a. Specifically, an Au electrode pattern was first deposited on a Si substrate through electron beam evaporation. Then, polystyrene (PS) was spin-coated on the surface of the Au pattern, and after drying, a thermal releasing tape (TRT) was applied onto the PS film to form Si/Au/PS/TRT structures. Subsequently, the fabricated Si/Au/PS/TRT structures were placed into deionized water to facilitate the separation of Au/PS/TRT from the Si substrate. Similarly, a MoS_2_ film grown on a sapphire substrate was also transferred to a TRT using the same method. This TRT, carrying the MoS_2_ film, was then placed onto an n-doped Si substrate pre-deposited with an Ag (or Cu) electrode (with Ti or Cr as an adhesion layer). The TRT was removed by heating, and the PS film was dissolved in toluene, thereby forming Si/Ag (or Cu)/MoS_2_ structures. Subsequently, the Au/PS/TRT stack was placed on the surface of the MoS_2_ film. Finally, the TRT was removed by baking, and the PS film covering the Au pattern was dissolved by immersing it in toluene, forming Si/Ag (or Cu)/MoS_2_/Au structures. Figures S3b and S3c show scanning electron microscopy (SEM) images of the fabricated Cu/MoS_2_/Au and Ag/MoS_2_/Au structures, respectively. An enlarged SEM image of the Ag/MoS_2_/Au structures is shown in Figure S3d, indicating the good continuity and integrity of the structures.

**
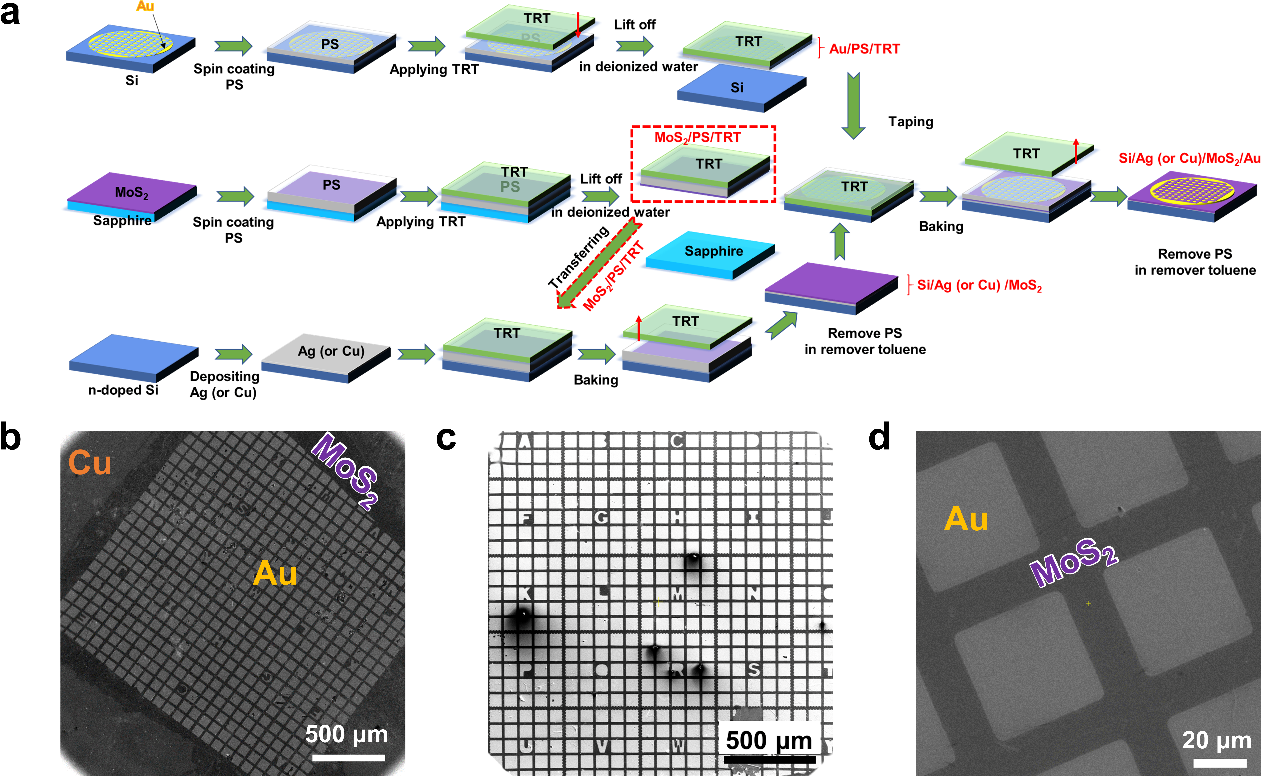
**

**Figure S3.** Fabrication of Ag (or Cu)/MoS_2_/Au sandwich structures. (a) Schematic illustration of the fabrication process for Ag (or Cu)/MoS_2_/Au sandwich structures. (b, c) SEM images of the fabricated Cu/MoS_2_/Au structures and Ag/MoS_2_/Au structures, respectively. (d) Enlarged SEM image of the Ag/MoS_2_/Au structures.

**S4. In situ construction of** **Ag (or Cu)/MoS_2_/W memristors.**

For in situ experiments, cross-sectional TEM samples were prepared by FIB. As schematically shown in Figure S4a, a Pt protection layer was initially deposited onto the n-doped Si /Ag (or Cu)/MoS_2_/Au structures. Corresponding cross-sectional samples were then acquired through ion beam milling and subsequently transferred onto a TEM grid. This TEM grid, with the attached sample, was then installed on one side of a TEM-STM holder. Subsequently, the Pt and Au protection layers were removed using a W tip mounted on the counter side of the holder. Figures S4b and S4c present the TEM images of the cross-sectional sample before and after the removal of the Pt and Au protection layers, respectively. Finally, the W tip was carefully manipulated to establish contact with the MoS_2_ film, thereby forming the Ag (or Cu)/MoS_2_/W memristors for in situ electrical characterization. A representative TEM image of the fabricated Ag/MoS_2_/W memristor is displayed in Figure S4d.

**
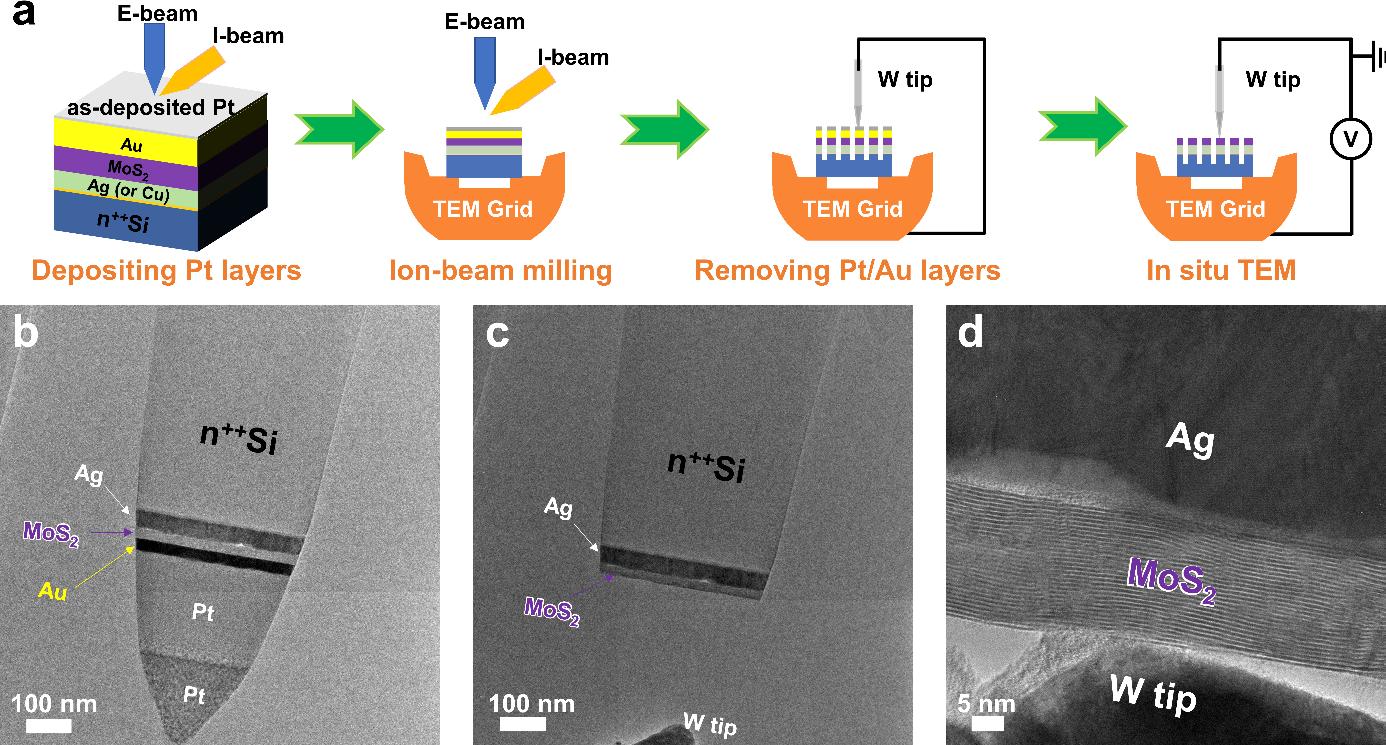
**

**Figure S4.** Fabrication of sample for in situ TEM. (a) Schematic illustration of the fabrication process for the sample used in the in situ TEM electrical measurements. (b) TEM image of a fresh sample after milling by FIB. (c) TEM image of the sample after removing the Au and Pt protection layers. (d) TEM image of the attachment of the W tip to the MoS_2_ layers, forming the Ag/MoS_2_/W structure.

**S5. CF growth in Cu/MoS_2_/W (or Pt) memristors.**

To further elucidate the growth direction of CFs in MoS_2_-based ECM memristors, Cu/MoS_2_/W memristors were constructed for in situ TEM experiments. Figures S5a-c show the corresponding time-sequence TEM images. As shown in Figure S5b, a CF (marked by a red dashed line) suddenly emerges. Upon continued voltage application, the size of this CF increases (Figure S5c). Meanwhile, a new CF (depicted by a blue dashed line) forms at the Cu electrode, suggesting that the growth direction of the CFs is from the anode (Cu) to the cathode (W).


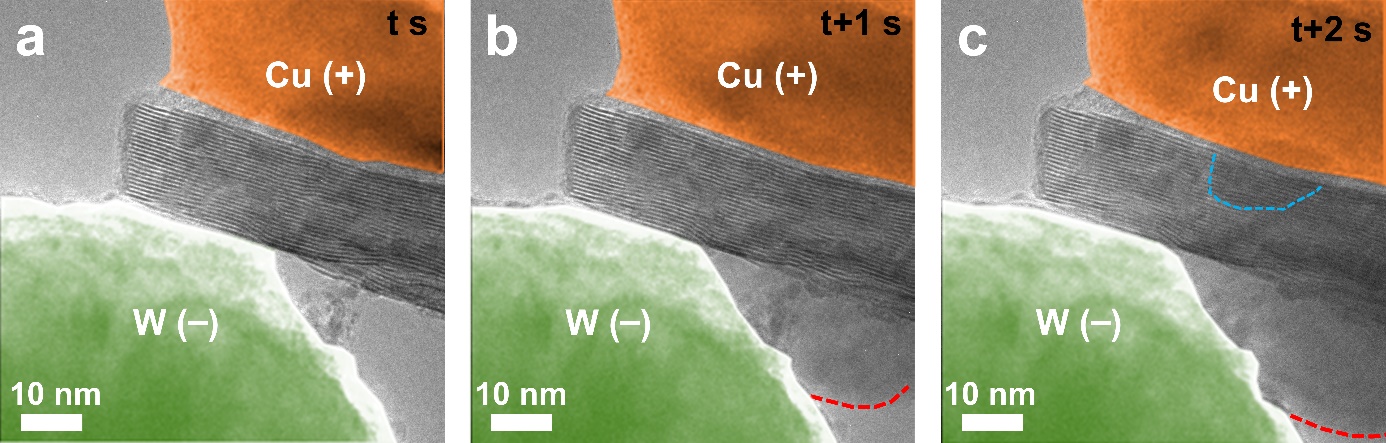


**Figure S5**. Time-sequence TEM images indicating the growth of the CFs formed from the Cu electrode to the W electrode in a Cu/MoS_2_/W memristor. The CFs are marked by red or blue dashed lines.

To more clearly elucidate the dynamic growth process of CFs, Cu/MoS_2_/Pt memristors were constructed (Figure S6a). The MoS_2_ was prepared by mechanical exfoliation, yielding superior crystallinity compared to that synthesized by the CVD method. This enhanced crystallinity is expected to slow the growth rate of CFs, thereby enabling clearer observation of their dynamic formation. After transferring MoS_2_ onto a Cu substrate, a Pt electrode was deposited using a FIB system. The Cu/MoS_2_/Pt memristors were then fabricated by FIB milling for in situ TEM experiments (Figure S6b). Figures S6c and S6d show CF growth after two successive voltage sweeps. After the first sweep, a CF (indicated by a red dashed line in Figure S6c) is observed extending from the Cu electrode. Following the second voltage sweep, this CF continues to grow towards the Pt electrode, while a new CF (highlighted by a blue dashed line in Figure S6d) also emerges from the Cu electrode. These results confirm that CF growth proceeds from the anode to the cathode.


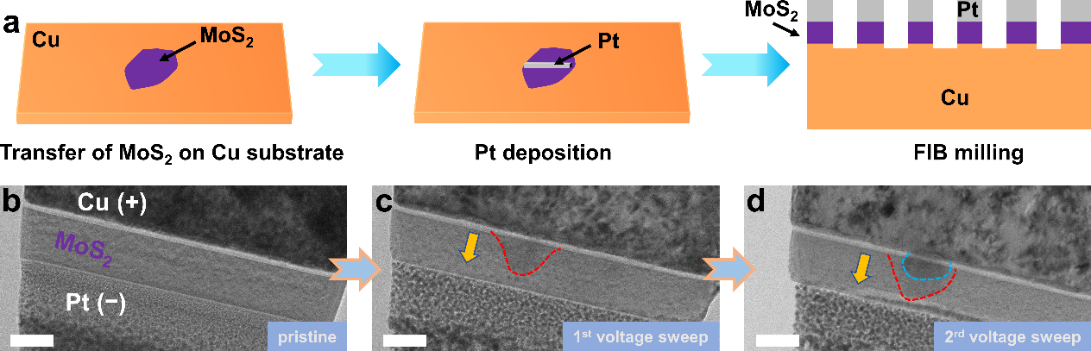


**Figure S6**. Growth of CFs in a Cu/MoS_2_/Pt memristor. (a) Schematic illustration of the Cu/MoS_2_/Pt memristor fabrication. (b) TEM image of a pristine Cu/MoS_2_/Pt memristor. (c, d) TEM images of the memristor after the first and the second voltage sweep, respectively. Scale bar: 50 nm.

**S6. Influence of electron beam.**

As shown in Figure S7a-c, no significant changes were observed in the MoS_2_ switching medium under continuous electron beam irradiation, and no CF formation occurred within the MoS_2_ layer. Moreover, an in situ experiment was conducted on an Ag/MoS_2_/W memristor with the electron beam switched off during electrical measurement. As shown in Figure S7d-f, under a positive voltage sweep (0 V→5 V→0 V), a CF (indicated by red dotted lines in Figure S7e) formed in the memristor, which induced the resistance switching behavior (Figure S7f). The results demonstrate that the electron beam irradiation does not influence CF formation, and the external electric field is the primary driver of CF growth.

**
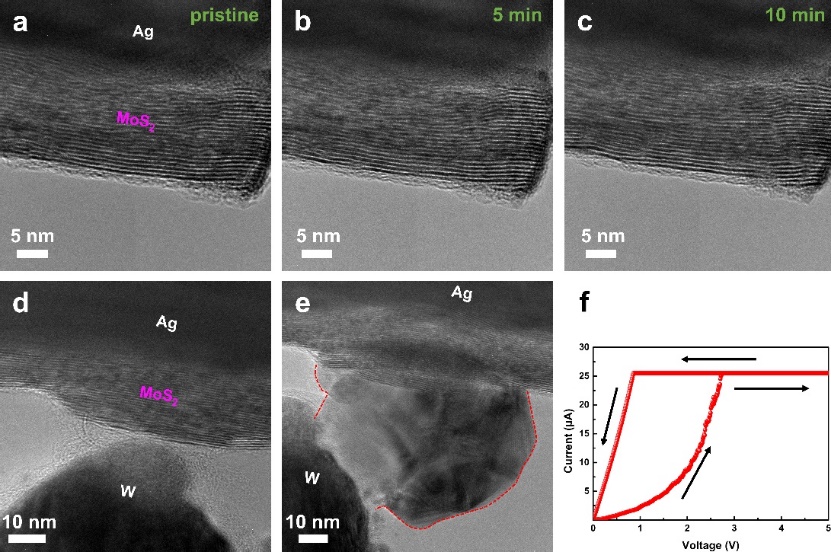
**

**Figure S7.** Influence of electron beam irradiation on the Ag/MoS_2_/W memristors. (a-c) TEM images of a pristine Ag/MoS_2_/W memristor (with the W tip extracted) and the device after electron beam irradiation for 5 min and 10 min, respectively. (d, e) TEM images of another Ag/MoS_2_/W memristor before and after the application of a voltage sweep (0 V→5 V→0 V). (f) *I-V* curve of the device. The arrows indicate the sweep direction.

**S7. Dissolution of CFs in the Cu/MoS_2_/W memristors.**

The dissolution process of the CF in a Cu/MoS_2_/W memristor was studied under a negative sweep voltage from 0 V to -1 V. As shown in Figure S8a-c, the size of the CF (marked by red dashed lines) becomes smaller and eventually disrupts at the CF/W interface. Meanwhile, the corresponding *I-V* curve exhibits an obvious decrease of electrical current when the voltage exceeds -0.52 V (Figure S8d).


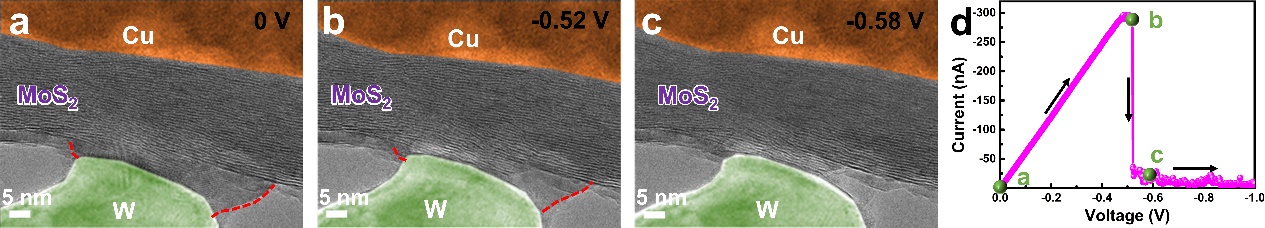


**Figure S8.** CF Dissolution in a Cu/MoS_2_/W memristor and the corresponding RS behavior. (a-c) TEM images of a Cu/MoS_2_/W memristor during the negative sweep voltage at 0 V, -0.52 V, -0.58 V, respectively. (d) *I-V* characteristics of the Cu/MoS_2_/W memristor under the negative voltage sweep.

Furthermore, to elucidate more details about the structure evolution of the MoS_2_ layer after the dissolution of CFs, HRTEM images of a Cu/MoS_2_/W memristor, acquired before and after CF dissolution were comparatively analyzed. Figure S9a shows a TEM image of the memristor in the LRS, which is also shown in Figure 2f. The blue rectangular region in Figure S9a is selected for an enlarged view (Figure S9b), where the MoS_2_ layer structure exhibits obvious deformation, and fringes ((111) planes of Cu) indicated by purple lines exist across the MoS_2_. Following CF dissolution, the distortion of MoS_2_ is substantially restored (Figure S9c), and the magnified image of the region framed by a yellow rectangle indicates that previous fringes have nearly disappeared (Figure S9d). Comparison of the TEM images before and after the CF dissolution demonstrates that filament removal can partially restore the MoS_2_ layer structure, indicating structural modification of the MoS_2_ channel.


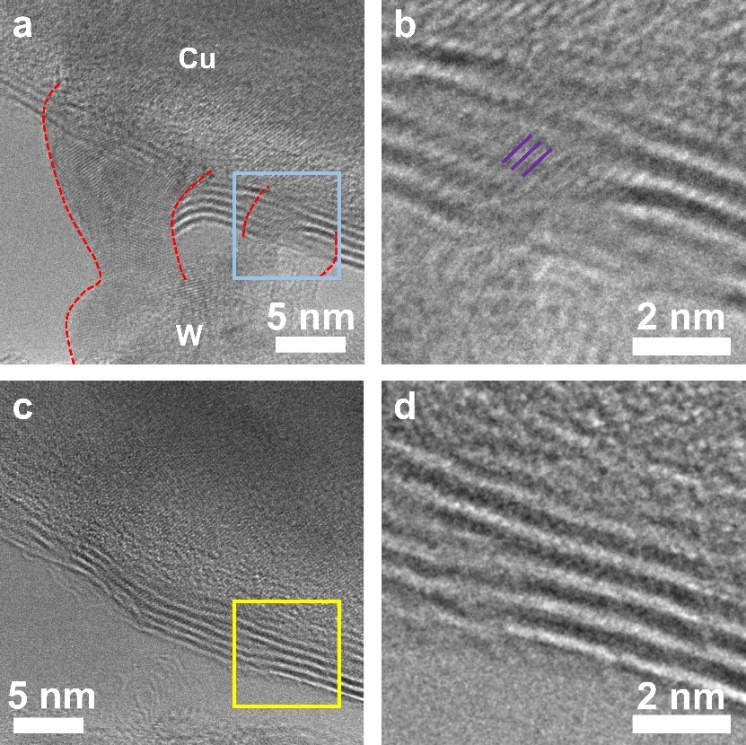


**Figure S9.** Comparison of MoS_2_ structure before and after CF dissolution. (a, c) TEM images of a Cu/MoS_2_/W memristor before and after CF dissolution, respectively. (b, d) Enlarged images of the regions outlined by blue and yellow rectangles in (a) and (c), respectively.

**S8. TEM Characterization of a fcc-Ag CF.**

Figure S10a shows the TEM image of an Ag/MoS_2_/W memristors after a SET operation, and the CF region framed by a red rectangle is selected for analysis. The corresponding HRTEM image and FFT pattern are shown in Figure S10b and S10c, respectively, confirming that the CF is composed of Ag with a fcc structure.


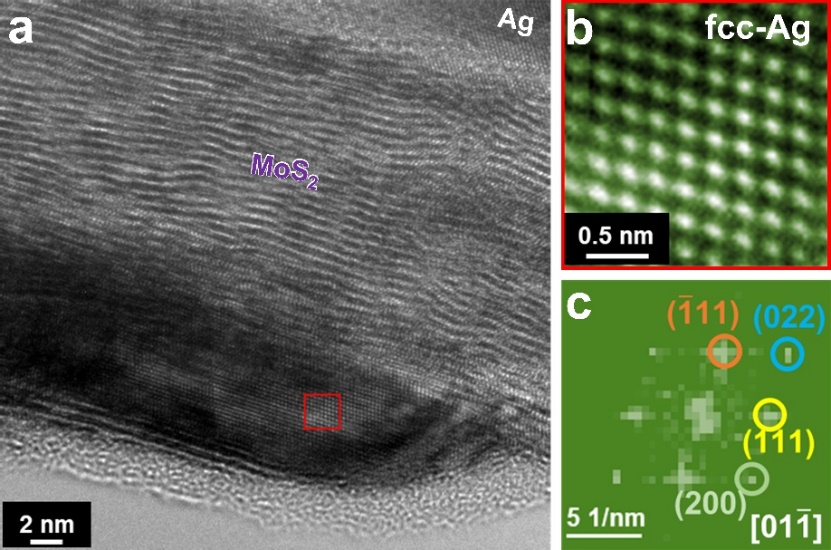


**Figure S10.** TEM characterization of the CF. (a) TEM image of a memristor after a SET operation. (b) HRTEM image of the region framed with the red box in (a), and the corresponding FFT pattern (c), indicating that the CF is composed of fcc structured Ag.

**S9. TEM Characterization of metallic sulfide-type CFs.**

Figure S11a-c show the TEM characterization of an Ag/MoS_2_/W memristor (with the W tip extracted) after a SET operation. The CF region framed by the red rectangle is selected for analysis. The corresponding HRTEM image and FFT pattern are shown in Figure S11b and S11c, respectively. The typical lattice spacings of 1.830 Å, 2.388 Å, and 2.212 Å can be indexed as the (13$\bar{\text{2}}$), ($\bar{\text{1}}$03), and (031) planes of monoclinic Ag_2_S along the [9$\bar{\text{1}}$3] zone axis, respectively, confirming that the CF is composed of Ag_2_S. Similarly, a Cu_2_S CF was found in a Cu/MoS_2_/W memristor, as shown in Figure S12a-c. After a negative voltage sweep, the CF nearly dissolved (Figure S12d).


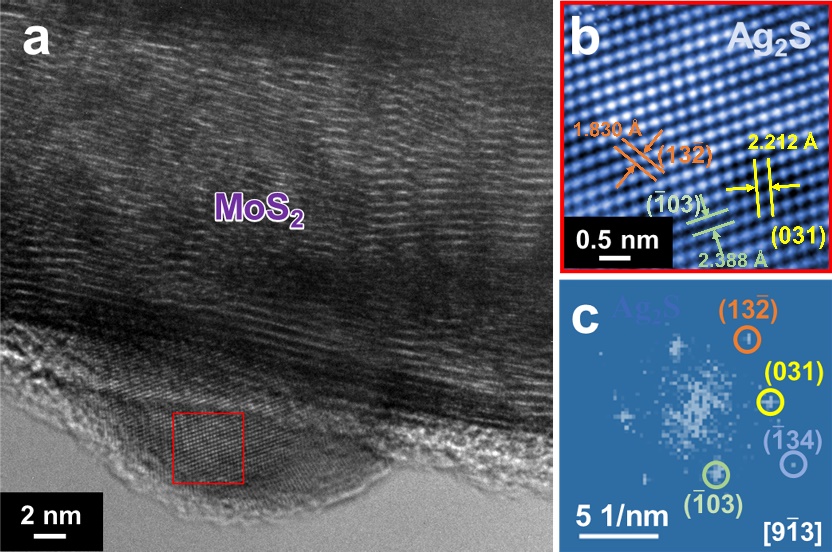


**Figure S11.** TEM characterization of an Ag_2_S CF. (a) TEM image of an Ag/MoS_2_/W memristor (with the W tip extracted) after the electroforming process. (b) HRTEM image of the region framed by the red box in (a), and the corresponding FFT pattern (c), indicating that the CF is composed of monoclinic Ag_2_S.


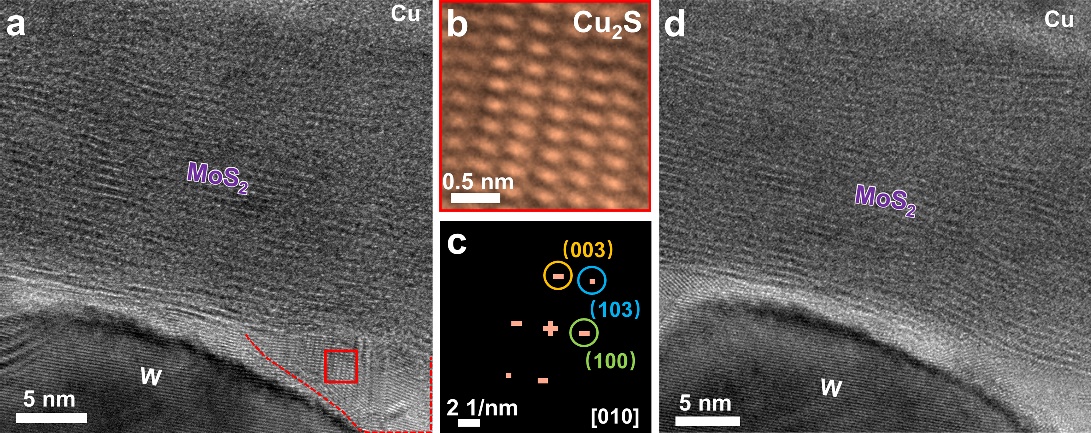


**Figure S12.** TEM characterization of a Cu_2_S CF. (a) TEM image of a Cu/MoS_2_/W memristor after CF formation. (b) HRTEM image of the CF region framed by the red box in (a), and the corresponding FFT pattern (c), confirming that the CF is composed of Cu_2_S. (d) TEM image of a Cu/MoS_2_/W memristor after CF dissolution.

**S10. Another reaction path for the formation of metal sulfide CFs.**

An alternative mechanism involves the migrating metal ions occupying molybdenum vacancies within MoS_2_ and subsequently forming chemical bonds with adjacent sulfur ions (Figure S13). The presence of inherent molybdenum vacancies in MoS_2_ readily accommodates the metal ions from the active metal electrode. Moreover, the growth of CFs can induce the formation of new molybdenum vacancies. Plausible routes for the creation of these new vacancies include the interaction of metal ions with the MoS_2_ lattice and local volumetric expansion within the MoS_2_ structure during the CF formation.

**
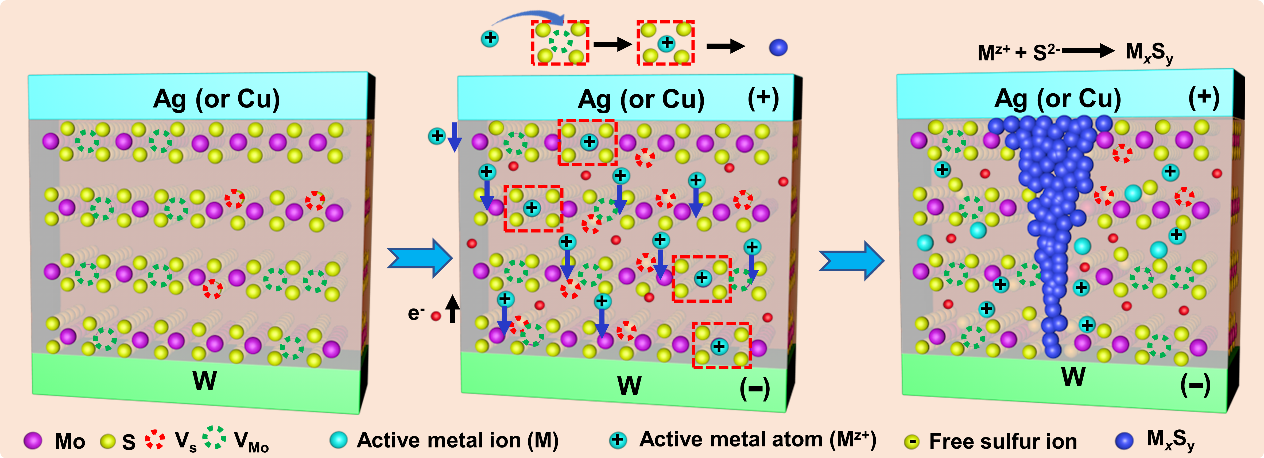
**

**Figure S13.** Schematic illustration of a possible formation mechanism of metal sulfide (M*_x_*S_y_) CFs, involving metal ions occupying molybdenum vacancies and subsequently reacting with neighboring sulfur ions.

To assess the energetic feasibility of metal sulfide formation resulting from metal ions occupying molybdenum vacancies within MoS_2_ and forming chemical bonds with neighboring sulfur ions, density functional theory (DFT) calculations were carried out using the Vienna Ab initio Simulation Package (VASP) with the projector augmented wave (PAW) method.^[1-4]^ The Perdew-Burke-Ernzerhof (PBE)^[5]^ functional with the generalized gradient approximation (GGA) method was used for the exchange-correlation functional.^[6]^ The cut-off energy of the plane-wave basis was set at 500 eV, and a k-mesh of 0.04×2π Å^-1^ was set for all samples. The self-consistent calculations applied a convergence energy threshold of 10^-5^ eV, and the equilibrium geometries were optimized with a maximum stress on each atom within 0.02 eV/Å. For the formation reaction, the formation energy (*E*_formation_) was calculated using the following equation:

$$\text{E}_{\text{formation}}=\text{E}_{\text{insertion}}-\text{E}_{\text{def}}-\text{E}_{\text{atom}}$$

Where *E*_insertion_ is the total energy of the insertion model, *E*_def_ is the total energy of the defective model, and *E*_atom_ is the energy of a single Ag/Cu atom obtained from metals.

DFT calculations indicate the formation energies of Ag-S and Cu-S chemical bonds are -0.2 eV and -1.19 eV in the MoS_2_ with molybdenum vacancies, respectively (Figure S14). These results confirm the energetic favorability for the formation of metal sulfide CFs via this pathway.


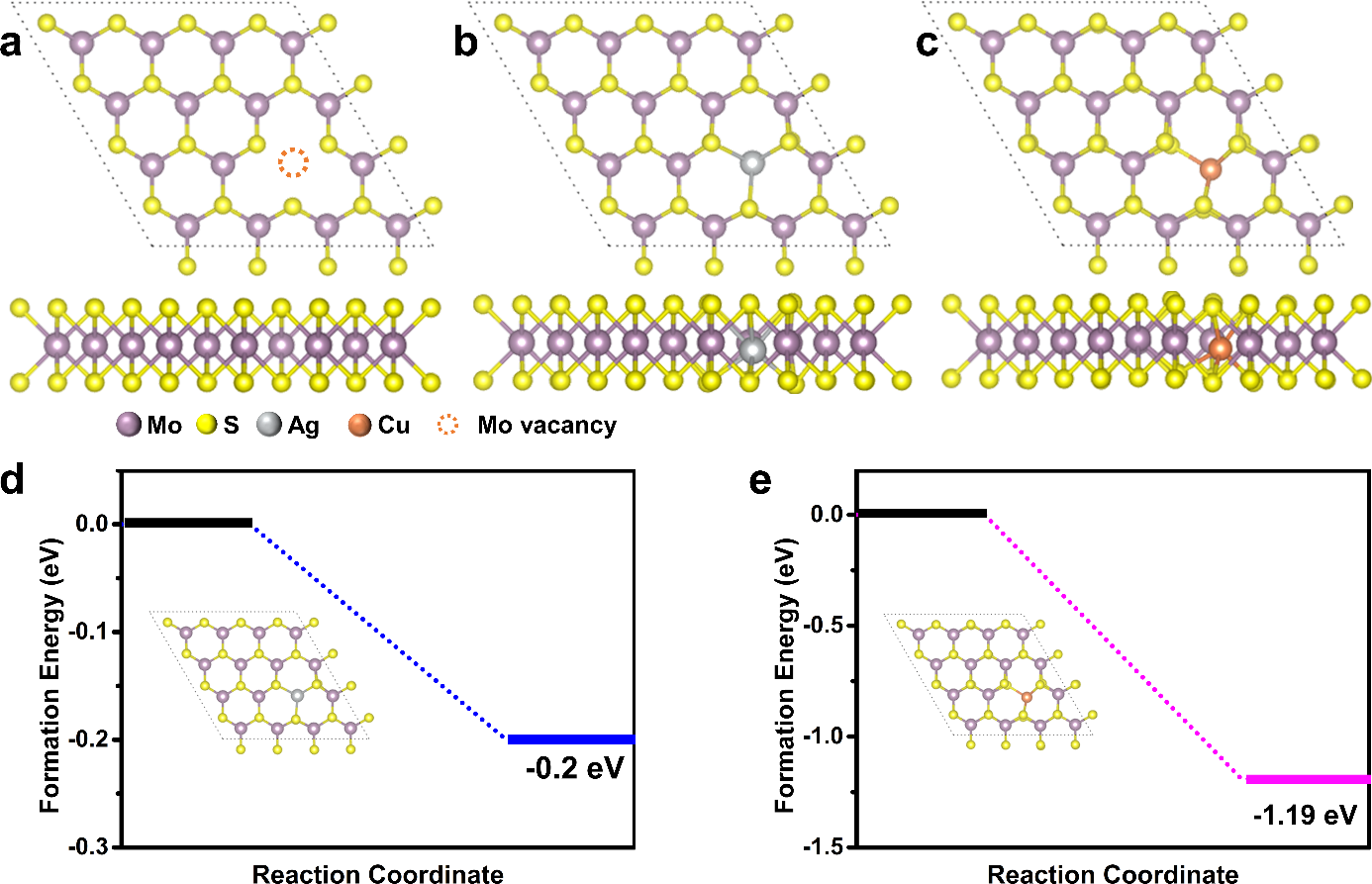


**Figure S14.** Formation energy of metal sulfides in MoS_2_ with a Mo vacancy. (a) Atom model of MoS_2_ with a Mo vacancy. (b, c) Atom models of MoS_2_ with Ag and Cu atoms occupying the Mo vacancy, respectively. (d, e) Formation energy of Ag-S and Cu-S bonds, respectively.

**References**

1. P. Hohenberg, W. Kohn, *Phys. Rev.* **1964**, *136*, B864.
2. W. Kohn, L. J. Sham, *Phys. Rev.* **1965**, *140*, A1133.
3. G. Kresse, J. Furthmüller, *Phys. Rev. B* **1996**, *54*, 11169.
4. P. E. Blöchl, *Phys. Rev. B* **1994**, *50*, 17953.
5. J. P. Perdew, K. Burke, M. Ernzerhof, *Phys. Rev. Lett.* **1996**, *77*, 3865.
6. S. Grimme, J. Antony, S. Ehrlich, H. Krieg, *J. Chem. Phys.* **2010**, *132*, 154104.
